# It’s not just the Therapist: Therapist and Country-Wide Experience Predict Therapist Adherence and Adolescent Outcome

**DOI:** 10.1007/s10566-016-9388-4

**Published:** 2017-01-06

**Authors:** Aurelie M. C. Lange, Rachel E. A. van der Rijken, Jan J. V. Busschbach, Marc J. M. H. Delsing, Ron H. J. Scholte

**Affiliations:** 1 Viersprong Institute for Studies on Personality Disorders, Kooikersweg 203 C, 5223 KE ‘s Hertogenbosch, The Netherlands; 2 Department of Psychiatry, Section of Medical Psychology & Psychotherapy, Erasmus Medical Center, Rotterdam, The Netherlands; 3 MST-Netherlands, Zevenbergen, The Netherlands; 4 Developmental Psychopathology, Behavioural Science Institute, Radboud University Nijmegen, Nijmegen, The Netherlands; 5 Praktikon, Nijmegen, The Netherlands

**Keywords:** Therapist adherence, Implementation, Multisystemic Therapy, Experience level, Treatment outcomes

## Abstract

**Objective:**

Therapist adherence is a quality indicator in routine clinical care when evaluating the success of the implementation of an intervention. The current study investigated whether therapist adherence mediates the association between therapist, team, and country-wide experience (i.e. number of years since implementation in the country) on the one hand, and treatment outcome on the other hand. We replicated and extended a study by Löfholm et al. (2014).

**Method:**

Data over a 10-year period were obtained from 4290 adolescents (12–17 years) with antisocial or delinquent problem behavior, who were treated with Multisystemic Therapy (MST) by 222 therapists, working in 27 different teams in the Netherlands. Multilevel structural equation modeling was used to assess the associations between experience, therapist adherence, and post-treatment outcomes.

**Results:**

Treatment outcomes were directly predicted by therapist experience, countrywide experience, and therapist adherence, but not by team experience. Moreover, therapist adherence mediated the association between therapist and country-wide experience, and treatment outcomes. The association between therapist experience and therapist adherence was not affected by the number of years of team experience or country-wide experience.

**Conclusion:**

The effect of country-wide experience on outcome may reflect increasing experience of training and supporting the therapists. It suggests that nation-wide quality control may relate to better therapist adherence and treatment outcome for adolescents treated with systemic therapy.

Evidence-based psychotherapies are being favored by many for their superior effectiveness over treatment as usual. However, the evidence supporting the effectiveness of these psychotherapies is often achieved in highly-controlled research settings (i.e. efficacy studies) that may not easily generalize to clinical practice (Henggeler 2011; Weisz et al. 2013). Indeed, studies conducted within everyday practice tend to achieve smaller effects than efficacy trials (Henggeler 2011). One of the reasons for this difference may be that it is harder for practitioners in everyday practice to deliver an evidence-based intervention as intended, i.e. to deliver the treatment with high adherence (Durlak and DuPre 2008; Shirk and Peterson 2013).

Therapist adherence is defined as the extent to which a therapist adheres to the treatment protocol or manual (McLeod et al. 2013; Perepletchikova and Kazdin 2005). Therapist adherence has been found to be related to positive treatment outcomes (Forgatch et al. 2005; Mihalic 2004; Schoenwald 2008). In addition, therapist adherence is a salient indicator of successful implementation (Durlak and DuPre 2008; Fixsen et al. 2005; McLeod et al. 2013; Schoenwald and Garland 2013). This is clearly represented in the implementation framework developed by Fixsen and colleagues (2005; see Fig. 1), in which therapist adherence is conceived as a part of treatment fidelity. The framework consists of several elements: the *source* represents the core components of the evidence-based intervention; the *destination* represents the practitioner who delivers the intervention; the *communication link* consists of practitioner training and coaching in order to maintain adherence to the core components. Since adherence is associated with treatment outcomes, the implementation framework also includes a *feedback* loop, which represents fidelity measures to monitor adherence at the level of the practitioner, the manager, and the organization. The implementation of an intervention may be influenced by factors other than just the practitioner, manager, and organization, as all interventions operate within a dynamic and demanding environment. This environment is visualized in the framework as *influence* and consists of factors such as funding, regulation, licensing, community relations, and agency collaboration (Durlak and DuPre 2008; Fixsen et al. 2005; Shirk and Peterson 2013).

**Fig. 1:**
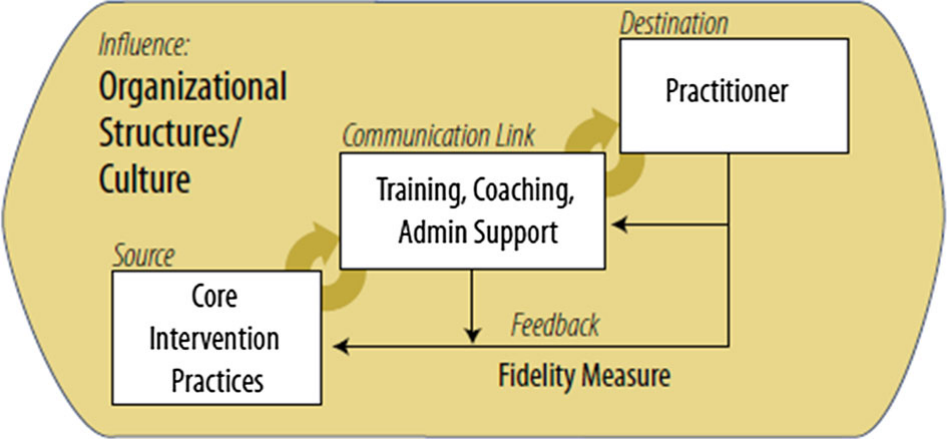
Implementation framework (Fixsen et al. 2005, p. 28)

Sustaining adequate adherence in clinical practice within this dynamic environment may be challenging (Durlak and DuPre 2008; Shirk and Peterson 2013). Several scholars have thus stressed the need to include quality-control methods (i.e. training, coaching, and monitoring instruments) to achieve and sustain adequate adherence and desirable outcomes (Garland and Schoenwald 2013; Henggeler and Sheidow 2012; Southam-Gerow and McLeod 2013). Without such quality-control methods, an intervention may quickly start to drift, resulting in lower adherence, reduced effectiveness, and inclusion of clients who do not meet the treatment’s inclusion criteria (Henggeler et al. 2008; Smith-Boydston et al. 2014).

An example of such an intervention with quality-control methods is Multisystemic Therapy (MST), an intensive home and community-based intervention addressing the multidetermined nature of the antisocial or delinquent behavioral problems of adolescents 12–18 years old (Henggeler et al. 2009). The MST quality-assurance system consists of data-driven and qualitative feedback loops to sustain adherence (Henggeler and Schoenwald 1999; Schoenwald 2008). It supports therapists and supervisors all over the world through the following: An initial training and quarterly booster sessions; weekly supervision and consultation on all cases; and data-driven monitoring of treatment outcomes and treatment adherence. Therapist adherence is monitored using the Therapist Adherence Measure (TAM; Henggeler and Borduin 1992). Organizational support is provided through manuals, initial meetings to prepare for the implementation of MST at the site, monitoring of program performance (such as therapist caseload and treatment duration), and continued expert consultation on program drift (internal and external factors affecting treatment fidelity; Henggeler and Schoenwald 1999; Schoenwald 2008). Research has shown that higher therapist adherence is associated with better treatment outcomes, such as favorable long-term criminal outcomes, fewer out-of-home placements, and better family functioning (e.g. Huey et al. 2000; Löfholm et al. 2014; Schoenwald et al. 2009a).

Nevertheless, even in the presence of a quality-assurance system, variance in therapist adherence occurs, a phenomenon which has also been observed within MST (MST Institute 2010, 2014). To date, little is known about the factors that influence therapist adherence. Identifying which factors affect therapist adherence is an essential step towards increasing the likelihood of successful implementation of interventions, leading to better treatment outcomes.

A recent study by Löfholm and colleagues (2014) suggested that therapist adherence may relate to the amount of experience of delivering a treatment. They studied therapist experience, team experience, and country-wide experience. Therapist experience did not predict therapist adherence, however team experience did predict adherence: Therapists achieved higher adherence scores when surrounded and supported by a team with more years of experience. Country-wide experience also predicted therapist adherence: Therapists who received training once the treatment had been running in the country for more than 2 years achieved higher adherence scores than ‘first-batch’ therapists, who started at the initial implementation of the treatment in the country. Trying to explain the absent relation between therapist experience and therapist adherence, Löfholm and colleagues hypothesized that therapist experience might start making a distinctive contribution only after the teams and organizations have acquired sufficient experience and stability to successfully support therapist adherence. The current study aimed to test this hypothesized moderating effect.

Because clinical implementation and treatment outcomes may be influenced by factors at different levels (i.e. youth and family, clinician, organization, and service system; Schoenwald 2008), cross-national replication of implementation research is important to assess the generalizability of previous findings. In fact, as health care systems, funding, regulation etc. may vary between countries, findings from one study are not necessarily applicable to other countries and settings. Therefore, we replicated the analytical model by Löfholm and colleagues (2014) using a longer follow-up period of Dutch MST data and extended the model by adding their hypothesized moderating effect. The present study tested (1) whether therapist and team experience with MST, and country-wide experience (i.e. time since implementation of MST) predicted therapist adherence; (2) whether therapist adherence predicted post-treatment outcomes (adolescent living at home, having had no police contact, and going to school or work); (3) whether the associations between therapist, team, and country-wide experience, and post-treatment outcomes were mediated by therapist adherence; and (4) whether the association between therapist experience and therapist adherence was moderated by team and country-wide experience. As such, we aimed to evaluate how experience with the treatment model at different levels (therapist, team, and country-wide) related to therapist adherence and treatment outcome.

## Method

### Participants and Procedures

#### Adolescents

A total of 5435 adolescents and their families completed MST between September 2004 and October 2014 in the Netherlands. Of these, 1145 families were excluded as they did not have any valid adherence assessments, resulting in a final sample of 4290 clients (79% of the total sample). No adolescents needed to be excluded due to missing data on the post-treatment outcomes, since completion of this information was a prerequisite for case closure.

The group excluded from analyses was compared with the sample included in the study (see Table 1). Adolescents excluded from analyses did not differ significantly from the study sample on sex, therapist experience, or team experience. However, the families excluded from the study were more likely to close their treatment due to lack of engagement or placement of the adolescent in a restrictive setting, and were more likely to have negative post-treatment outcomes. Adolescents excluded from the study were also older than adolescents in the study sample, and therapists treating these excluded adolescents started earlier in the implementation process than therapists treating included families. No information was available on ethnicity or socioeconomic status of the families.

**Table 1:** Descriptive statistics for families included in and excluded from the study sample

	Families included in study	Families excluded from study	*p*
*N*	*4290*	*1145*	
*Dichotomous variables*	*%*	*%*	
Gender (male)	70%	69%	.80
Interview language (Dutch)	87%	–	–
Home	91%	80%	.00
School/Work	83%	75%	.00
No new arrests	86%	82%	.00
*Continuous variables*	*M (SD)*	*M (SD)*	
Age	15.62 (1.38)	15.78 (1.34)	.00
Therapist experience	16.37 (13.78)	16.23 (15.35)	.78
Team experience	3.22 (2.23)	3.10 (2.70)	.17
Country-wide experience	3.71 (2.20)	3.11 (2.25)	.00
Therapist adherence	4.32 (0.53)	–	–

#### Therapists and Teams

Initially, 4 Dutch MST teams, divided over 2 organizations, started providing MST. During the period under study, the number of teams grew to 27 divided over 8 organizations. Two teams switched to another organization during the course of this study. These teams were considered as the same team over the whole period of data collection.

In total, 222 therapists and 48 supervisors, supervised by 13 consultants, provided MST over the course of this study. Although no information was available about their level of education, therapists should have completed higher education in a relevant domain to qualify for MST therapist in the Netherlands.

#### Procedures

Families were referred to MST due to severe externalizing behavioral problems of the adolescent, such as delinquency, problems at school, or risk of out-of-home placement. Families had to meet the MST inclusion criteria, which have been specified by MST Services, the international licensor for the dissemination of MST (MST Services 2014). Case enrollment and discharge information, as well as the TAM/TAM-R were entered into the MST Institute website (MSTi; www.MSTInstitute.org) by a staff person working in the organization that housed the MST team. The collected information from MSTi consisted of start and end date of the treatment, TAM interview language, gender and date of birth of the adolescent, and therapist-reported post-treatment outcomes. As this study used data retrospectively, formal consent was not required.

### Measures

All measures in the current study were defined in the same way as in the original study by Löfholm and colleagues (2014). In cases where this was not possible, this is explicitly mentioned.

#### Therapist Adherence

The original Therapist Adherence Measure (TAM; Henggeler and Borduin, 1992) was developed to monitor therapist adherence to the MST model and consisted of 26 items rated on a 5-point Likert scale (1 = *not at all*, 2 = *a little*, 3 = *some,* 4 = *pretty much*, and 5 = *very much*). The TAM was scored by the primary caregiver who was called on a monthly basis by an agency staff other than the family’s therapist. Items assessed therapist adherence to the MST clinical process and the treatment principles of MST, such as ‘The therapist tried to understand how my family’s problems all fit together’ and ‘The therapist’s recommendations required family members to work on our problems almost every day’.

Later improvements of the TAM led to inclusion of an additional 12 items assessing whether the treatment focused on important aspects of the adolescent’s school, peer, and neighborhood, consistent with the MST model. Psychometric analyses of this new set of items resulted in a revised instrument, the Therapist Adherence Measure-Revised (TAM-R; Henggeler et al. 2006a), consisting of 19 of the original 26 items and 9 of the additional 12 items (Schoenwald et al. 2008a). These 28 items were rated on the same 5-point Likert scale as the original TAM.

Predictive validity and reliability of the TAM and TAM-R were assessed during initial randomized clinical trials of MST, as well as later transportability studies (Henggeler et al. 1997, 2002; Schoenwald et al. 2003, 2008a). Discriminant validity was supported by findings that the TAM discriminated between MST and treatment as usual (Henggeler et al. 2006b).

The Dutch TAM was introduced in the Netherlands after translation and back-translation by two independent translation offices and after approval by MST Services at the end of 2004. The revised TAM-R was introduced in 2007. The English and Dutch TAM-R can be found on http://www.mstinstitute.org/qa_program/tam_languages.shtml. Since the TAM-R was introduced halfway the period under study, the analyses were conducted on the 19 items overlapping in both the TAM and TAM-R. In the current study, the average therapist-adherence score for each family based on these 19 items correlated highly (*r* = .99) with the average therapist-adherence score per family based on all 28 items.

Only valid assessments (assessments with a maximum of three missing items, and where face-to-face contact between the family and the therapist had occurred in the last 2 weeks prior to administration of the TAM or TAM-R) were included for analyses. The mean number of adherence-reports provided by the caregivers was 3.5 (*SD* = 1.6). Since TAM ratings have been found to be stable within a family’s treatment episode (Schoenwald 2008) and the internal consistency of the 19 items was high (α = .92), a mean adherence-score could be calculated for each family. This score represented the mean of all completed reports by the family during an MST treatment episode and represented the mean level of therapist adherence as experienced by this family.

#### Country-Wide Experience

Country-wide experience was defined as the number of years since MST had been implemented in the Netherlands at the time the therapist first started providing MST. This variable was represented at the cohort level (see data analysis strategy below for a definition of cohort). It was comparable to the dichotomous variable ‘wave’ in the Swedish study by Löfholm and colleagues (2014), the only difference being that our variable was continuous instead of binary. Since the implementation of MST in the Netherlands was not characterized by two consecutive waves, as was the case in Sweden, the current continuous operationalization was chosen. As a 10-year period was used in the present study, country-wide experience ranged from 0 to 9 years.

#### Team Experience

Team experience was defined as the number of years a team had been active in providing MST at the time the family began treatment. Team experience ranged from 0 to 9 years.

#### Therapist Experience

Therapist experience was defined as the number of previous families to whom the therapist had provided MST. This score ranged from 0 to 81 families.

#### TAM Interview Language

MST was provided to families with divergent ethnic backgrounds, including families for whom Dutch was not their first language. In these cases the TAM/TAM-R could be administered in another than the Dutch language. Language was dichotomized in Dutch or a different language. If interview language had varied within a family over the course of treatment, a family was categorized as Dutch only if all of the TAM/TAM-R administrations had been in Dutch.

#### Post-Treatment Outcomes

Post-treatment outcomes were reported by the therapist at the completion of treatment and consisted of three dichotomous outcomes, namely whether the adolescent (a) lived at home (i.e. all stable home situations, including, but not restricted to, living with grand parents or foster parents), (b) was engaged in school (no truancy) or work (at least 20 h a week), and (c) had not been arrested. The first two outcomes represented the situation at the end of treatment. However, if the adolescent was placed out of home during treatment, MST was stopped, leading to a negative treatment outcome for this MST episode. The third outcome referred to all arrests during the MST treatment episode. These three outcomes are being used by MST as ultimate outcomes and should be attained by all adolescents at the end of treatment (MST Institute 2016). All three outcomes have been operationalized and standardized by MST Services to ensure that these outcomes are being scored in the same way by all therapists. As MST is a community-based treatment, MST therapists have connections with all relevant youth services in their working area, and can easily request information to validate their scores.

### Data Analyses Strategy

Analyses were performed in Mplus 7.3 (Muthén and Muthén 1998–2012) for multilevel structural equation modeling. The amount of missing data was minimal (gender: 5%; language: 5%) and were taken into account using robust full maximum likelihood (MLR). Also any deviates from normality were addressed with MLR, as MLR is a robust estimator for non-normal and dependent data using all available data.

The model consisted of three levels: Families (level 1; N=4290) were nested within ‘cohorts’ (all the families seen by the same therapist in the same ‘team-experience’ year; level 2; N=816), and cohorts were nested within therapists (level 3; N=222). Cohorts were included as a level as it was assumed that families treated by the same therapist in the same year would be more similar than families treated by the same therapist a couple of years later, when therapist and team experience would have increased. To account for the non-independence in the data the TYPE = COMPLEX TWOLEVEL command in Mplus was used (Asparouhov and Muthén 2006; Muthén and Muthén 1998–2012). The COMPLEX command was used to adjust standard errors for non-independence within therapists. As such, non-independence within therapists was accounted for but not explicitly modeled. In contrast, non-independence within cohorts was explicitly included into the model using the TWOLEVEL command.

#### Analytical Model

**Fig. 2:**
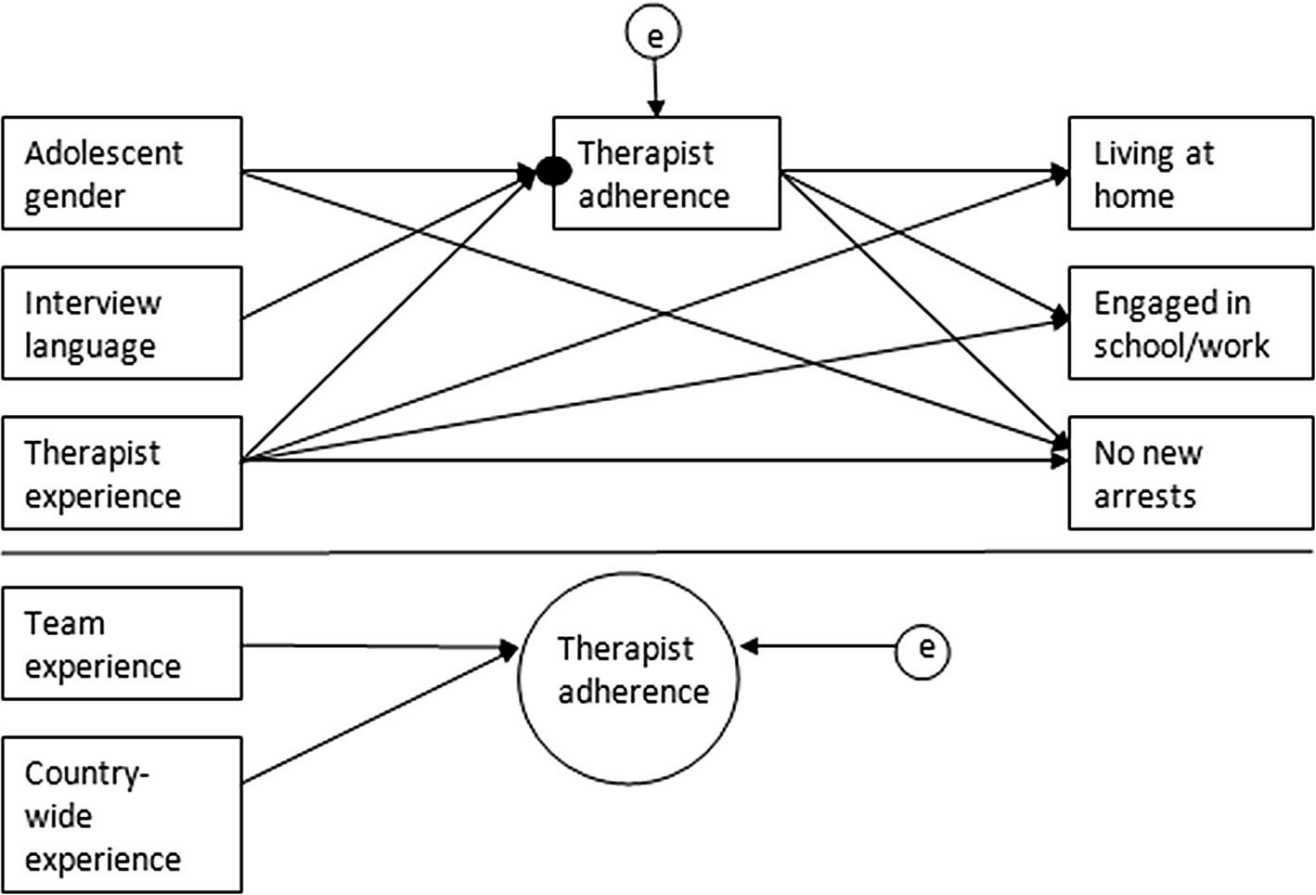
Visual representation of original model. Variables above the line were measured at the family level, whereas years of team experience was measured at the cohort level. The small filled circle represents the random intercept of therapist adherence, which was allowed to vary across cohorts. At the cohort level, therapist’s yearly TAM/TAM-R is the TAM intercept-as-outcome. Circles with the letter ‘e’ are error terms that represent unexplained variance. The cohort-level error term for therapist adherence represents unexplained variance in its random intercept

The first aim of this study was to replicate the analytical model of Löfholm and colleagues (2014) and investigate the associations of therapist, team, and country-wide experience with therapist adherence and post-treatment outcomes. The model was specified as follows (see Fig. 2). At the family level (level 1), therapist experience, adolescent gender, and TAM-interview language were included as predictors of therapist-adherence scores (the mean therapist adherence level achieved in a family). Therapist adherence in turn was included as a predictor for the three post-treatment outcomes (adolescent living at home, engaged in school/work, and no new arrests). As the post-treatment outcomes were categorical, the resulting model was a logistic regression model. A direct path was specified from adolescent gender to ‘no new arrests’, and from therapist experience to all three post-treatment outcomes. Further, the model allowed the intercept of therapist adherence to vary across cohort clusters. At the cohort level (level 2), team experience and country-wide experience were included as predictors of the intercept of therapist adherence. The adherence intercept represented the therapist’s overall adherence score for all the families seen by that therapist in a single year of team experience. An additional 39 cohorts were created to account for cross-classified therapists (i.e. therapists that were members of two teams in that year).We also included direct paths from team experience and country-wide experience to the three treatment outcomes, and used the Akaike Information Criterion (AIC) and the Bayesian Information Criterion (BIC) for model selection. Rules of thumb suggest that an increase between four and seven for each additional parameter on the AIC and an increase between two and six for each additional parameter on the BIC may be positive evidence for the alternative model (Burnham and Anderson 2004; Raftery 1999).

#### Mediation Analyses

We subsequently tested for indirect effects of therapist, team, and country-wide experience on post-treatment outcomes via therapist adherence. These analyses were only conducted if the individual paths from experience to adherence, and from adherence to outcome were significant. Indirect effects were identified using the joint significance test (MacKinnon et al. 2002). This test consists of adding the joint paths of experience-to-adherence and adherence-to-outcome to the model, and evaluates whether the combination of both these paths is significant. If these joint paths are significant, the association between experience and outcome is mediated through therapist adherence.

#### Moderator Analyses

Lastly, we extended the model with two cross-level interactions to investigate whether team or country-wide experience moderate the association between therapist experience and therapist adherence. These cross-level interactions were added to the model independently of one another, by adding a path from team and country-wide experience to the slope from therapist experience to therapist adherence. The AIC and BIC were used for model selection (Burnham and Anderson 2004; Raftery 1999).

## Results

Table 2 provides an overview of the development of the therapist-adherence scores and post-treatment outcomes over the course of the study. Adherence scores and post-treatment outcomes increased in the first few years and then appeared to stabilize.

**Table 2:** Development of adherence scores and post-treatment outcomes over time

Year of implementation	Year 1&2	Year 3&4	Year 5&6	Year 7&8	Year 9&10
*N*	182	704	1075	1270	1059
Mean and *SD* of therapist adherence (19 items)	3,93 (0.47)	4,35 (0.49)	4,36 (0.51)	4,31 (0.55)	4,34 (0.53)
Home	84%	89%	91%	92%	92%
School/work	77%	81%	83%	84%	84%
No new arrests	75%	83%	84%	86%	92%

### Preliminary Family-Level Model

First, we evaluated a single-level model, which consisted of only the family-level paths, to gain an initial idea about the Chi square-based model fit at the family level prior to building a multilevel model. Model fit was evaluated using a robust weighted least-squares estimator (weighted least squares mean and variance adjusted; Muthén and Muthén 1998–2012). Although the Chi square value was significant (χ^2^ = 14.75, *p* = .04), indicating bad model fit, all other model fit indices met the criteria for good fit (CFI = .99, TLI = .96, RMSEA = .02). As the Chi square value is sensitive to large sample-sizes, and all other model fit indices were good, we assumed good model fit for the single-level model.

### Analytical Model

#### Predictors of Therapist Adherence

Table 3 presents the parameter estimates for family- and cohort-level paths specified in the model. At the family-level, therapist adherence was predicted by therapist experience and language, but not the gender of the adolescent. Therapist adherence was higher when the therapist had more experience and the TAM-R was assessed in another than the Dutch language. On average, therapist-adherence scores increased with 1.25% (an increase of 0.05 on a scale of 1 to 5) for an additional ten families treated (approximately one additional year of experience). At the cohort-level, country-wide experience predicted therapist adherence. A therapist starting 1 year later in the implementation process acquired therapist-adherence scores that were 0.5% higher (an increase of 0.02) than therapists starting 1 year earlier in the implementation process.

**Table 3:** Parameter estimates original model for 19-item adherence scores

Outcome	Predictor	Estimate	95% CI	*p*	Standardized effect
Level 1					
Therapist adherence	Therapist experience	0.005	[0.002, 0.007]	.00	0.14
	Female	0.03	[−0.01, 0.06]	.16	0.02
	Dutch language	−0.07	[−0.12, −0.02]	.00	−0.05
Home	Therapist adherence	0.40	[0.24, 0.56]	.00	0.11
	Therapist experience	0.003	[−0.005, 0.01]	.48	0.02
School/work	Therapist adherence	0.58	[0.42, 0.73]	.00	0.15
	Therapist experience	0.006	[−0.001, 0.01]	.10	0.04
No new arrests	Therapist adherence	0.29	[0.11, 0.46]	.00	0.07
	Female	0.97	[0.70, 1.24]	.00	0.24
	Therapist experience	0.01	[0.002, 0.02]	.02	0.08
Level 2					
Mean yearly therapist adherence	Team experience	−0.02	[−0.03, 0.001]	.06	−0.18
	Country-wide experience	0.02	[0.004, 0.03]	.02	0.20

Parameter estimates for therapist adherence are linear regression coefficients. Estimates for post-treatment outcomes (home, school/work, no new arrests) are logistic regression coefficients. Standardized effects for continuous predictors are path coefficients standardized with respect to both predictor and outcome. Standardized effects for dichotomous predictors are path coefficients standardized with respect to the outcome only

#### Predictors of Post-Treatment Outcomes

All three post-treatment outcomes were predicted by therapist adherence (see Table 3). This indicates that higher therapist adherence increased the odds that the adolescent was living at home at the end of the treatment (*OR* = 1.49, 95% CI [1.27,1.74]), that the adolescent was engaged in school or work (*OR* = 1.78, 95% CI [1.53, 2.06]), and that the adolescent had not been arrested during the course of the treatment (*OR* = 1.33, 95% CI [1.12, 1.58]). As causality could be reversed, with behavioral change during treatment leading to higher therapist adherence instead of adherence leading to better treatment outcomes, we conducted a sensitivity analysis to see whether early treatment therapist adherence similarly predicted treatment outcome. For this purpose, we replaced the mean therapist adherence score in the model by adherence as assessed during the first month of MST. This did not change any of the model results. Therapist adherence increased the odds that the adolescent was living at home at the end of treatment (*OR* = 1.69, 95% CI [1.46, 1.94]), that the adolescent was engaged in school or work (*OR* = 1.79, 95% CI [1.55, 2.06]), and that the adolescent had not been arrested during the course of the treatment (*OR* = 1.28, 95% CI [1.10, 1.49]).

Higher therapist experience only increased the odds for not having been arrested (*OR* = 1.01, 95%CI [1.00, 1.02]). An odds ratio of 1.01 for ‘no new arrests’ indicated that a 1-unit increase in therapist experience (one additional family treated) was associated with a 1% increase in the odds that the adolescent had not been arrested during the course of the treatment. Thus, after approximately 1 year of additional therapist experience (ten additional families treated) the odds of ‘no new arrests’ increased with 11%. Moreover, girls had higher odds of not having been arrested during the course of the treatment than boys (*OR* = 2.63, 95% CI [2.01, 3.44]).

We also tested the direct effect of team experience and country-wide experience on post-treatment outcomes. AIC and BIC substantially improved when including those direct paths (∆ AIC = 92, ∆ BIC = 34, ∆ df = 11). Results are presented in Table 4. Team experience only predicted ‘engaged in school/work’ (*B* = −0.07, *p* < .01), indicating that one additional year of team experience was associated with a 7% decrease in the odds that the adolescent was engaged in school or work. Country-wide experience significantly predicted ‘engaged in school/work’ (*B* = 0.09, *p* < .001) and ‘no new arrests’ (*B* = 0.16, *p* < .01). One additional year of country-wide experience was associated with a 9% increase in the odds that the adolescent was engaged in school/work, and a 16% increase in the odds that the adolescent had no new arrests.

**Table 4:** Parameter estimates expanded model, including direct effects on outcome on Level 2

Outcome	Predictor	Estimate	95% CI	*p*	Standardized effect
Level 1					
Therapist adherence	Therapist experience	0.004	[0.002, 0.007]	.00	0.13
	Female	0.02	[−0.01, 0.06]	.21	0.02
	Dutch language	−0.07	[−0.12, −0.03]	.00	−0.05
Home	Therapist adherence	0.40	[0.23, 0.56]	.00	0.11
	Therapist experience	0.01	[−0.003, 0.02]	.09	0.08
School/work	Therapist adherence	0.58	[0.42, 0.73]	.00	0.15
	Therapist experience	0.02	[0.01, 0.03]	.00	0.12
No new arrests	Therapist adherence	0.26	[0.08, 0.44]	.01	0.07
	Female	1.02	[0.75, 1.33]	.00	0.25
	Therapist experience	0.02	[0.01, 0.03]	.00	0.13
Level 2					
Therapist adherence	Team experience	−0.01	[−0.03, −0.002]	.08	−0.16
	Country-wide experience	0.02	[0.004, 0.03]	.02	0.20
Home	Team experience	−0.05	[−0.11, 0.02]	.15	−0.21
	Country-wide experience	0.08	[−0.02, 0.18]	.10	0.33
School/work	Team experience	−0.07	[−0.12, −.02]	.00	−0.38
	Country-wide experience	0.09	[0.05, 0.14]	.00	0.47
No new arrests	Team experience	0.03	[−0.03, 0.08]	.36	0.08
	Country-wide experience	0.16	[0.06, 0.26]	.00	0.46

Parameter estimates for therapist adherence are linear regression coefficients. Estimates for post-treatment outcomes (home, school/work, no new arrests) are logistic regression coefficients. Outcomes at level 2 were mean yearly scores. Standardized effects for continuous predictors are path coefficients standardized with respect to both predictor and outcome. Standardized effects for dichotomous predictors are path coefficients standardized with respect to the outcome only

### Mediation Analyses

Indirect effects of experience on post-treatment outcomes through therapist adherence were tested for therapist experience and country-wide experience. We did not include team experience in these analyses as team experience did not significantly predict therapist adherence. Monte Carlo confidence intervals were computed using the web utility of Selig and Preacher (2008), as these non-symmetric intervals are more appropriate when the assumption of a normal distribution for the indirect effects may not hold (Preacher and Selig 2012). Indirect effects of therapist experience were significant for all three post-treatment outcomes (*B* = .002, 95% CI [0.000, 0.003], *p* < .05 for ‘living at home’; *B* = .003, 95% CI [0.001, 0.004], *p* < .01 for ‘engaged in school/work’; *B* = .001, 95% CI [0.000, 0.002], *p* < .05 for ‘no new arrests’). Indirect effects of country-wide experience were only significant for ‘living at home’ (*B* = .008, 95% CI [0.001, 0.016], *p* < .05) and ‘engaged in school/work’ (*B* = .01, 95% CI [0.002, 0.022], *p* < 001.05), but not for ‘no new arrest’ (*B* = .005, 95% CI [0.001, 0.012], *p* = .07).

### Moderator Analyses

Team experience and country-wide experience were independently included as a moderator of the slope from therapist experience to therapist adherence. AIC and BIC values were higher when including the moderator paths, indicating worse fit. Inspection of these models showed that residual variance of the slope was zero, indicating that there was no random effect of therapist experience on therapist adherence. Therefore we concluded that the effect of therapist experience on therapist adherence was equal across the whole range of team experience and country-wide experience. Thus, we did not find a moderator effect of team or country-wide experience on the association between therapist experience and adherence.

## Discussion

The current study replicated the mediation model of Löfholm and colleagues (2014), examining whether (1) therapist, team, and country-wide experience predicted therapist adherence, (2) therapist adherence predicted post-treatment outcomes (adolescent living at home, having had no police contact, and going to school or work), and (3) the associations between therapist, team, and country-wide experience, and post-treatment outcomes were mediated by therapist adherence. Moreover, we extended the model with a moderator path, resulting in a fourth question: We investigated whether the association between therapist experience and therapist adherence was moderated by team and country-wide experience. Our findings indicate that (1) therapist and country-wide experience, but not team-experience, predict therapist adherence; (2) therapist adherence predicts all three post-treatment outcomes; (3) therapist adherence also mediates the associations between therapist and country-wide experience on the one hand, and treatment outcome on the other hand, and (4) the association between therapist experience and therapist adherence is not moderated by team or country-wide experience. Contrary to the study by Löfholm and colleagues (2014), we found therapist experience instead of team experience to predict therapist adherence and treatment outcome. The role of country-wide experience in our study was similar to its role in the Swedish study: More country-wide experience is associated with higher therapist adherence, and therapist adherence mediates the effects of country-wide experience on post-treatment outcomes.

The finding that country-wide experience is a significant predictor of therapist adherence and treatment outcome corresponds with previous studies underscoring the relevance of outer contextual factors for implementation of evidence-based interventions. Such factors include interorganizational networks, connections with evidence-based intervention developers (Fixsen et al. 2005; Novins et al. 2013), but also the use of quality-control methods, such as the MST quality-assurance system (Henggeler et al. 2008; Holth et al. 2011; Smith-Boydston et al. 2014). Methods such as adherence monitoring, training, and supervision may be especially relevant, as these are directly targeted at sustaining adequate therapist adherence and positive treatment outcomes over time (Bond et al. 2014; Fixsen et al. 2005; Garland and Schoenwald 2013; Novins et al. 2013). Moreover, these methods may be particularly sensitive to country-wide experience. The director of MST-the Netherlands, the Dutch Network Partner licensed to implement MST, has suggested that, over the years, MST-the Netherlands has gained a better understanding of the core components of MST and has improved its ability to deploy the quality-assurance system (personal communication with Wim van Geffen, June 2015).

This study suggests that differences in therapist-adherence scores between different countries, as have been found for MST adherence scores (Lange et al. 2015; MST Institute 2010), may partly be attributable to differences in country-wide experience of supporting adherence. Nevertheless, one must bear in mind that many factors may affect therapist adherence scores. For example, cultural differences in response style have been proposed as an alternative explanation for the differences observed between Dutch and American adherence scores (Lange et al. 2015). As the current study was not designed to provide evidence for the origin of these differences, it does not allow drawing any definite conclusions on the role of country-wide experience in this.

Unlike the Löfholm et al. study (2014) report, we found that therapist experience was a significant predictor of therapist adherence. Our findings are similar to recent analyses of 2298 MST therapists worldwide, showing that therapists with more MST experience achieved higher adherence scores than therapists with less experience (MST Institute 2014). Nevertheless, the effect of therapist experience on adherence was small in our study. This may be a consequence of the overall high adherence scores, an effect also reported in the study by Löfholm and colleagues (2014). This left little room for these scores to improve any further, which may explain the absence of a significant effect in the Löfholm report.

The association between therapist experience and therapist adherence was not moderated by country-wide experience. This may be due to a similar ceiling effect of therapist-adherence scores as described above. If so, studies reporting a wider range of adherence scores, for example due to implementation difficulties, may be better capable of illuminating these associations.

Contrary to the study by Löfholm and colleagues (2014), team experience did not predict therapist adherence in our study. Although, so far, we remain uncertain as to the cause of this difference, various explanations are possible. For example, turnover of therapists, supervisors, or consultants may have been greater in the Dutch teams than in the Swedish ones. Thus, it may not be the experience of the team, but rather the experience of the supervisor which is predictive of therapist adherence. Previous research has shown how supervisor adherence is related to therapist adherence, suggesting that supervisor behavior is related to therapist behavior (Schoenwald et al. 2009b). Another unexpected finding was the negative association between team experience and the engagement of the adolescent in school or work. As this was the only significant association of team experience, this may be a Type I error. However, the association between team experience and therapist adherence was also negative, albeit non-significant (*p*=.06). Anecdotal evidence suggests that, at least in some teams, more team experience may lead supervisors and their teams to pay less attention to the adherence scores, which may result in lower therapist adherence and subsequently lower treatment outcomes.

### Limitations

We have suggested that the effect of country-wide experience on therapist adherence and treatment outcome may reflect increasing experience of training and supporting MST therapists, indicating successful implementation of MST. Nevertheless, therapist adherence is only one aspect of treatment fidelity. Assessing a broader range of treatment fidelity measures may provide a more complete picture of how experience is related to successful implementation. For example, Brunk et al. (2014) have developed an index of treatment fidelity for MST, including not only measures of therapist adherence, but also items on critical program practices, such as organizational support and essential clinical operations. Future research may benefit from using such an index.

The families excluded from our study differed with regard to some key variables from the families included for study. Most notably, excluded families had poorer post-treatment outcomes than the study sample, and MST had been implemented for a shorter period of time when their therapists were engaged as an MST therapist. Therefore, we hypothesize that excluded families reported lower therapist adherence scores than included families. This may have restricted the range of adherence scores in our study and, as such, hampered the likelihood of finding strong effects. However, we have no reason to believe it influenced the direction of our effects.

As we did not have information regarding behavioral change during treatment, we cannot rule out the possibility that initial behavioral change led to higher therapist adherence scores instead of the adherence predicting the positive treatment outcomes. Nevertheless, we have several indications to assume that the direction of the effects is as discussed. We felt safe using average family adherence scores because these scores have been found to be stable within families (Schoenwald 2008a). The sensitivity analysis showed that early treatment adherence similarly predicted treatment outcomes. Moreover, the results of this study are in line with numerous previous studies that demonstrated the association between adherence and treatment outcome within MST (e.g. Huey et al. 2000; Löfholm et al. 2014; Schoenwald et al. 2009a).

The aim of the current study was to replicate previous findings to investigate whether these findings are robust across different countries that are characterized by different health care settings, funding agencies etc.. Schoenwald and colleagues (2008b) have described how MST needs to be adapted at several levels to be suitable for implementation in other countries. As a consequence, findings from one country cannot be assumed to apply to other countries. Replicating studies across countries can help researchers and practitioners understand what factors affect implementation success and, more specifically, therapist adherence. This study confirmed previous findings on the relevance of country-wide experience, but also calls for more research into the role of team experience, as the findings of team experience in relation to therapist adherence and outcome were opposite to previous research.

## Conclusions

This study showed that therapist experience as well as country-wide experience matters for sustaining therapist adherence and achieving favorable treatment outcomes. Implementing an intervention with high adherence is not an easy task; stakeholders at different levels need to acquire experience in delivering the intervention with high adherence or in supporting therapists to do so. Using a quality-assurance system may be essential to sustaining therapist adherence and warranting good treatment outcomes.

## References

[CR1] Asparouhov, T., & Muthén, B. (2006). Multilevel modeling of complex survey data. In *Proceedings of the joint statistical meeting in seatle, August 2006. ASA Section on Survey Research Methods*. (pp. 2718–2726).

[CR2] Bond GR, Drake RE, McHugo GJ, Peterson AE, Jones AM, Williams J (2014). Long-term sustainability of evidence-based practices in community mental health agencies. Administration and Policy In Mental Health.

[CR3] Brunk MA, Chapman JE, Schoenwald SK (2014). Defining and evaluating fidelity at the program level in psychosocial treatments: A preliminary investigation. Zeitschrift für Psychologie.

[CR4] Burnham KP, Anderson DR (2004). Multimodal inference. Understanding AIC and BIC in model selection. Sociological Methods and Research.

[CR5] Durlak JA, DuPre EP (2008). Implementation matters: A review of research on the influence of implementation on program outcomes and the factors affecting implementation. American Journal of Community Psychology.

[CR6] Fixsen, D. L., Naoom, S. F., Blase, K. A., Friedman, R. M., & Wallace, F. (2005). *Implementation research: A synthesis of the literature.* Tampa, FL: University of South Florida, Louis de la Parte Florida Mental Health Institute, The National Implementation Research Network (FMHI Publication #231).

[CR7] Forgatch MS, Patterson GR, DeGarmo DS (2005). Evaluating fidelity: Predictive validity for a measure of competent adherence to the Oregon model of parent management training. Behavior Therapy.

[CR8] Garland A, Schoenwald SK (2013). Use of effective and efficient quality control methods to implement psychosocial interventions. Clinical Psychology: Science and Practice.

[CR9] Henggeler S (2011). Efficacy studies to large-scale transport: The development and validation of Multisystemic Therapy programs. Annual Review of Clinical Psychology.

[CR10] Henggeler, S. W., & Borduin, C. M. (1992). *Multisystemic therapy adherence scale.* Unpublished instrument. Charleston, SC: Department of Psychiatry and Behavioral Sciences, Medical University of South Carolina.

[CR11] Henggeler SW, Schoenwald SK (1999). The role of quality assurance in achieving outcomes in MST programs. Journal of Juvenile Justice and Detention Services.

[CR12] Henggeler SW, Sheidow AJ (2012). Empirically supported family-based treatments for conduct disorder and delinquency in adolescents. Journal of Marital and Family Therapy.

[CR13] Henggeler SW, Melton GB, Brondino MJ, Scherer DG, Hanley JH (1997). Multisystemic Therapy with violent and chronic juvenile offenders and their families: The role of treatment fidelity in successful dissemination. Journal of Consulting and Clinical Psychology.

[CR14] Henggeler SW, Schoenwald SK, Liao JG, Letourneau EJ, Edwards DL (2002). Transporting efficacious treatments to field settings: The link between supervisory practices and therapist fidelity in MST programs. Journal of Clinical Child Psychology.

[CR15] Henggeler SW, Borduin CM, Schoenwald SK, Huey SJ, Chapman JE (2006). MST therapist adherence measure-revised (TAM-R).

[CR16] Henggeler SW, Halliday-Boykins CA, Cunningham PB, Randall J, Shapiro SB, Chapman JE (2006). Juvenile drug court: Enhancing outcomes by integrating evidence-based treatments. Journal of Consulting and Clinical Psychology.

[CR17] Henggeler SW, Sheidow AJ, Cunningham PB, Donohue BC, Ford JD (2008). Promoting the implementation of an evidence-based intervention for adolescent marijuana abuse in community settings: Testing the use of intensive quality assurance. Journal of Clinical Child and Adolescent Psychology.

[CR18] Henggeler SW, Schoenwald SK, Borduin CM, Rowland MD, Cunningham PB (2009). Multisystemic therapy for antisocial behavior in children and adolescents.

[CR19] Holth P, Torsheim T, Sheidow AJ, Ogden T, Henggeler S (2011). Intensive quality assurance of therapist adherence to behavioral interventions for adolescent substance use problems. Journal of Child and Adolescent Substance Abuse.

[CR20] Huey SJ, Henggeler SW, Brondino MJ, Pickrel SG (2000). Mechanisms of change in Multisystemic therapy: Reducing delinquent behavior through therapist adherence and improved family and peer functioning. Journal of Consulting and Clinical Psychology.

[CR21] Lange AM, Scholte RH, van Geffen W, Timman R, Busschbach JJ, van der Rijken REA (2015). The lack of cross-national equivalence of a therapist adherence measure (TAM-R) in Multisystemic Therapy (MST). European Journal of Psychological Assessment.

[CR22] Löfholm CA, Eichas K, Sundell K (2014). The Swedish implementation of Multisystemic Therapy for adolescents: Does treatment experience predict treatment adherence?. Journal of Clinical Child and Adolescent Psychology.

[CR23] MacKinnon DP, Lockwood CM, Hoffman JM, West SG, Sheets V (2002). A comparison of methods to test mediation and other intervening variable effects. Psychological Methods.

[CR24] McLeod BD, Southam-Gerow MA, Tully CB, Rodriguez A, Smith MM (2013). Making a case for treatment integrity as a psychosocial treatment quality indicator for youth mental health care. Clinical Psychology: Science and Practice.

[CR25] Mihalic S (2004). The importance of implementation fidelity. Report on Emotional and Behavioral Disorders in Youth.

[CR26] MST Institute (2010). *2010 MST Data Report.* Retrieved July 2015, from http://www.mstinstitute.org.

[CR27] MST Institute (2014). *2014 MST Data overview report.* Retrieved July 2015, from http://www.mstinstitute.org.

[CR28] MST Institute (2016). *Frequently Asked Questions. MST Institute Enhanced Website*. Retrieved May 2016, from https://www.msti.org/documents/EW_FAQs.pdf.

[CR29] MST Services (2014*). Multisystemic Therapy*^*®*^*(MST*^*®*^*) Organizational Manual*. Charleston, SC: Author.

[CR30] Muthén, L., & Muthén, B. O. (1998–2012). *Mplus user’s guide. Seventh Edition.* Los Angeles, CA: Author.

[CR31] Novins DK, Green AE, Legha RK, Aarons GA (2013). Dissemination and implementation of evidence-based practices for child and adolescent mental health: A systematic review. Journal of the American Academy of Child and Adolescent Psychiatry.

[CR32] Perepletchikova F, Kazdin AE (2005). Treatment integrity and therapeutic change. Issues and research recommendations. Clinical Psychology: Science and Practice.

[CR33] Preacher KJ, Selig JP (2012). Advantages of Monte Carlo confidence intervals for indirect effects. Communication Methods and Measures.

[CR34] Raftery AE (1999). Bayes factors and BIC. Sociological Methods and Research.

[CR36] Schoenwald SK (2008). Toward evidence-based transport of evidence-based treatments: MST as an example. Journal of Child and Adolescent Substance Abuse.

[CR37] Schoenwald SK, Garland AF (2013). A review of treatment adherence measurement methods. Psychological Assessment.

[CR38] Schoenwald SK, Sheidow AJ, Letourneau EJ, Liao JG (2003). Transportability of Multisystemic Therapy: Evidence for multilevel influences. Mental Health Services Research.

[CR39] Schoenwald SK, Carter RE, Chapman JE, Sheidow AJ (2008). Therapist adherence and organizational effects on change in youth behavior problems one year after Multisystemic Therapy. Administration and Policy in Mental Health and Mental Health Services Research.

[CR40] Schoenwald SK, Heiblum N, Saldana L, Henggeler SW (2008). The international implementation of Multisystemic Therapy. Evaluation and the Health Professions.

[CR41] Schoenwald SK, Chapman JE, Sheidow AJ, Carter RE (2009). Long-term youth criminal outcomes in MST transport: The impact of therapist adherence and organizational climate and structure. Journal of Clinical Child and Adolescent Psychology.

[CR42] Schoenwald SK, Sheidow AJ, Chapman JE (2009). Clinical supervision in treatment transport: Effects on adherence and outcomes. Journal of Consulting and Clinical Psychology.

[CR43] Selig, J. P., & Preacher, K. J. (2008). Monte Carlo method for assessing mediation: An interactive tool for creating confidence intervals for indirect effects [Computer software]. Available from http://quantpsy.org/.

[CR44] Shirk SR, Peterson E (2013). Gaps, bridges, and the bumpy road to improving clinic-based therapy for youth. Clinical Psychology: Science and Practice.

[CR45] Smith-Boydston JM, Holtzman RJ, Roberts MC (2014). Transportability of Multisystemic Therapy to community settings: Can a program sustain outcomes without MST Services oversight?. Child & Youth Care Forum.

[CR46] Southam-Gerow MA, McLeod BD (2013). Advances in applying treatment integrity research for dissemination and implementation science: Introduction to special issue. Clinical Psychology: Science and Practice.

[CR47] Weisz JR, Ugueto AM, Cheron DM, Herren J (2013). Evidence-based youth psychotherapy in the mental health ecosystem. Journal of Clinical Child and Adolescent Psychology.

